# Trust as moral currency: Perspectives of health researchers in sub-Saharan Africa on strategies to promote equitable data sharing

**DOI:** 10.1371/journal.pdig.0000551

**Published:** 2024-09-27

**Authors:** Qunita Brown, Jyothi Chabilall, Nezerith Cengiz, Keymanthri Moodley

**Affiliations:** 1 Department of Medicine, Division for Medical Ethics and Law, Faculty of Medicine and Health Sciences, Stellenbosch University, Cape Town, South Africa; 2 Business Management, Faculty of Medicine and Health Sciences, Stellenbosch University, Cape Town, South Africa; Fundación Progreso y Salud: Junta de Andalucia Consejeria de Salud y Familias Fundacion Progreso y Salud, SPAIN

## Abstract

Groundbreaking data-sharing techniques and quick access to stored research data from the African continent are highly beneficial to create diverse unbiased datasets to inform digital health technologies and artificial intelligence in healthcare. Yet health researchers in sub-Saharan Africa (SSA) experience individual and collective challenges that render them cautious and even hesitant to share data despite acknowledging the public health benefits of sharing. This qualitative study reports on the perspectives of health researchers regarding strategies to mitigate these challenges. In-depth interviews were conducted via Microsoft Teams with 16 researchers from 16 different countries across SSA between July 2022 and April 2023. Purposive and snowball sampling techniques were used to invite participants via email. Recorded interviews were transcribed, cleaned, coded and managed through Atlas.ti.22. Thematic Analysis was used to analyse the data. Three recurrent themes and several subthemes emerged around strategies to improve governance of data sharing. The main themes identified were (1) Strategies for change at a policy level: guideline development, (2) Strengthening data governance to improve data quality and (3) Reciprocity: towards equitable data sharing. Building trust is central to the promotion of data sharing amongst researchers on the African continent and with global partners. This can be achieved by enhancing research integrity and strengthening micro and macro level governance. Substantial resources are required from funders and governments to enhance data governance practices, to improve data literacy and to enhance data quality. High quality data from Africa will afford diversity to global data sets, reducing bias in algorithms built for artificial intelligence technologies in healthcare. Engagement with multiple stakeholders including researchers and research communities is necessary to establish an equitable data sharing approach based on reciprocity and mutual benefit.

## Author summary

In our study, we explore the challenges and potential solutions for data sharing among health researchers in sub-Saharan Africa (SSA). While data sharing can significantly enhance the development of digital health technologies and artificial intelligence by providing diverse and unbiased datasets, many researchers in SSA are cautious about sharing their data. Through in-depth interviews with 16 researchers from different countries in SSA, we identified key strategies to overcome these challenges. Our findings highlight three main areas: the need for policy changes to develop clear guidelines, the importance of strengthening data governance to improve data quality, and the value of reciprocity to ensure equitable data sharing. Trust is a critical component in promoting data sharing, which can be built by enhancing research integrity and governance at both micro and macro levels. High-quality data from Africa can contribute to more diverse global datasets, helping to reduce bias in healthcare algorithms.

## Background

Recent data sharing and collaborative research in the African region demonstrates a bold move away from the exploitative research processes of the past towards the promotion of more equitable research strategies [1–3]. Researchers, especially within the health sector, are encouraged to engage in data sharing activities once key management processes are in place [4,5].

Data sharing is seen as logical knowledge progression contributing to scientific innovation not only in the primary research field, but also for secondary research when anonymised data is used for further scientific enquiry [6]. Furthermore, African data is important to contribute to improving the diversity of global databases [7]. This contribution is essential to avoid algorithmic discrimination that creates bias in machine learning for the development of digital health technologies [1]. While data sharing may not be a prevailing tradition among SSA researchers—who have been victims of research exploitation for decades—African research itself has not been stymied [8–11]. One of the more recent examples of exploitation in the context of data sharing amongst collaborating research institutions involved the Wellcome Sanger Institute in the UK and some South African universities [8,12]. Allegations were raised around the unconsented use of African genomic data to develop commercial genomic products [8–12]. Recently, researchers in SSA have been advocating for transparency, trust and fairness in the context of dependable collaborations [11,13,14].

Africa is constituted of mainly low and middle-income countries (LMICs) many of which still need to develop institutional or national data sharing policies and guidelines [3,15]. Some African countries have developed legislation based on foreign laws such as the European Union General Data Protection Act (EU-GDPR) (2018) [16,17]. South Africa applies the Protection of Personal Information Act (POPIA) (2020) [18] seeking to achieve impartial stringent governance systems [19,20]. Other research-intensive countries in Africa that provide data protection laws ranging from moderate to strict include Nigeria, Kenya, Ethiopia and Uganda [21]. However, there are some African countries that do not have robust legislation [3]. This variability in legislation from one country to the next creates a degree of insecurity amongst researchers with respect to the level of protection afforded by laws and guidelines [3,15].

Consequently, African researchers have been apprehensive to share their data if data sharing agreements, acceptable administration and trained support staff are deficient [22]. Researchers interviewed in SSA countries as part of our parent study identified several challenges associated with sharing their data. There was individual researcher concerns associated with fears regarding data sharing. Academic pressure to publish exacerbated such data sharing fears. Furthermore, structural issues linked to data collection and storage in SSA impacted data quality and sharing [23]. Researchers indicated that recognition in academia sometimes led to “scooping” of research data [23]. Ethical challenges experienced by researchers in SSA included concerns around confidentiality and informed consent primarily linked to risk of sharing with others [23]. Commercialisation and benefit sharing also created ethical dilemmas for researchers [23]. Finally, legal challenges imply suboptimal governance processes that impacted negatively on willingness to share data [23]. This paper takes those challenges into account and addresses the research gap resulting from the paucity of literature from the Global South reflecting perspectives of researchers on opportunities and strategies to address the challenges.

In the sections that follow, the study design and sampling, data collection process, analysis, and ethical considerations are outlined. This is followed by the presentation of the study results that illuminate themes and subthemes derived from thematic analysis. The final sections include the discussion, strengths, and limitations. We offer recommendations and concluding thoughts.

## Materials and methods

### Study design and sampling

This study is linked to a previous descriptive, cross-sectional, online survey of researchers from SSA countries conducted to provide broad context to issues around data sharing in SSA [15]. Respondents were invited to anonymously participate through Research Electronic Data Capture (REDCap). The findings of the quantitative study are reported and discussed in the paper “Data sharing and data governance in sub-Saharan Africa: Perspectives from researchers and scientists engaged in data-intensive research” authored by Kabanda et al. (2023) [15]. The sample consisted of 160 researchers and scientists representing 43 sub-Saharan African countries and was conducted between June 2022 to September 2022 [15]. The study presented and reported on demographic information, data use among respondents, data practices, data management support, data sharing and data protection [15].

Of those participants from the quantitative study who agreed to be interviewed, anonymised branching logic was used to redirect them from REDCap to a Google Form. At that point, they were able to provide their email addresses in which we used to send consent forms for the qualitative study.

Using a qualitative study design, we further explored researchers’ views on data sharing and its related challenges and opportunities across SSA by conducting 16 online in-depth interviews (IDIs). From a methodological perspective conducting the interviews provided us the opportunity to triangulate the findings from the quantitative aspect of the study.

A mixture of purposive and snowball sampling was used to recruit research participants. More specifically, potential research participants were identified through existing networks of the Division for Medical Ethics and Law. We also conducted some web searches with the aim of recruiting researchers that are working in the data intensive health research sphere. Regarding the snowball sampling, following the completion of the interviews, participants were asked by interviewers if they were prepared to ascertain whether any of their colleagues would be interested in participating in the research. Researchers had to ensure that these new participants were suitable in terms of the study criteria. Interested parties then contacted the interviewer to schedule interviews. Participants were invited to participate in the study in their personal capacity on a voluntary basis.

### Data collection

Between July 2022 and April 2023, 16 in-depth interviews were conducted in English via Microsoft Teams. Interviews lasted an average of 45 minutes and consent to record each interview was obtained. The interview guide was developed based on a literature review and expert consultation with colleagues from the School for Data Science and Computational Thinking, Stellenbosch University. The interview guide comprised of five sections which delved into participants’ professional background; their experiences and understanding of data sharing; thoughts on the benefits and challenges of data sharing; knowledge of existing guidelines and frameworks related to data sharing; and recommendations on the development of a data sharing guideline policy (S1 File). After 16 interviews, data saturation was reached at a continental level as participants expressed similar challenges and strategies to resolve these.

### Data analysis

Audio files were cleaned and transcribed verbatim. In terms of validation, the audio recordings were cross checked with the transcripts to ensure accuracy. Atlas.ti (version 22) was used to manage the data. Transcripts were thematically coded. The thematic method proposed by Braun and Clarke (2006) was used for data analysis [24]. The six steps of the iterative process are useful in identifying common patterns in qualitative datasets [24]. Due to the flexible nature of thematic analysis, it has been used to analyse large sets of qualitative data as well as smaller samples of one to two participants [24].

Each interview was coded independently by one researcher using a combination of inductive and deductive reasoning for theme development. While the deductive analysis traced the themes listed in the interview guide, the inductive analysis allowed for further expansion. Major themes of interest were identified and categorised, followed by an in-depth analysis of the themes through discussion among the team of involved researchers. Adjustments to the final thematic map were made to improve logical cohesion. Intercoder reliability was also established once 20% of randomly selected transcripts were independently coded by two researchers.

### Ethical aspects

The in-depth interviews posed minimal risk as the sample included educated and empowered respondents who had full capacity to consent or decline participation. Ethics approval was granted by the Faculty of Medicine and Health Sciences Health Research Ethics Committee (Reference No: N22/03/028) at Stellenbosch University, South Africa.

## Results

A total of 16 researchers from 16 SSA countries participated in the study. Most researchers are actively involved in mixed methods research within the public health sector, they also contribute to teaching and practicing medicine. In terms of experience, there was a mixture of early career, mid-career, and researchers with an extensive background in health research (See Table 1).

**Table 1:** Breakdown of respondents.

Participant	Role	Age Category	Type of Research	Methodology (Research)
IDI 1	Researcher	30–39	Public Health	Quantitative
IDI 2	Computer Scientist, Researcher, Lecturer	40–49	Public Health	Mixed Methods
IDI 3	Researcher	30–39	Geospatial and Public Health	Mixed Methods
IDI 4	Researcher	30–39	Public Health	Mixed Methods
IDI 5	Researcher, Lecturer	50–59	Public Health	Mixed Methods
IDI 6	Medical doctor, Researcher	30–39	Public Health	Mixed Methods
IDI 7	Researcher	50–59	Public Health	Quantitative
IDI 8	Lawyer, Researcher	30–39	Public Health	Mixed Methods
IDI 9	Researcher, Lecturer	40–49	Public Health	Mixed Methods
IDI 10	Bioinformatician, Researcher	30–39	Public Health	Quantitative
IDI 11	Researcher, Lecturer	40–49	Public Health	Mixed Methods
IDI 12	Researcher	30–39	Public Health	Mixed Methods
IDI 13	Medical doctor, Researcher	40–49	Public Health and Policy	Mixed Methods
IDI 14	Medical virologist, Researcher	50–59	Public Health	Mixed Methods
IDI 15	Researcher	60–69	Public Health	Mixed Methods
IDI 16	Pharmacokineticist, Researcher, Lecturer	50–59	Public Health	Quantitative

Countries represented in the sample included Mauritius, Burundi, Democratic Republic of Congo (DRC) [25] and 13 Anglophone countries—Botswana, Ethiopia, Eswatini, Ghana, Kenya, Malawi, Namibia, Nigeria, South Africa, Tanzania, Uganda, Zambia, and Zimbabwe [26,27]. The sample largely comprised Anglophone countries given that the research team is English speaking. The five most research-intensive countries in SSA in the fields of public health, environmental and occupational health are included in the sample -South Africa, Nigeria, Kenya, Uganda and Ethiopia [21].

The community of researchers working with large data sets in SSA is currently limited and was limited at the time interviews were conducted. This community faces similar challenges and despite borders, the responses were similar as the interviews proceeded. Interviews continued until data saturation was reached. This was validated in the transcriptions and analysis when the quotes were becoming repetitive. The major difference is that some countries have stronger legislation than others. The research-intensive countries tend to have data protection legislation in place as they engaged in collaborative research with high income countries that do have such legislation.

Three salient themes emerged within the context of strategies to promote data sharing. The findings are presented and discussed through the lens of an updated perspective of the ecological systems theory originally developed by Bronfenbrenner in the early 1970’s [28]. Bronfenbrenner’s theoretical framework has proved to be valuable across different disciplines [29,30] notably in social work practice, where the use of concepts such as micro-, meso- and macro-systems are frequently used [31]. A multi-level approach to presenting and interpreting the findings was constructively adopted to demonstrate the interrelated relationships between the strategies proposed by the researchers from SSA [32]. Moreover, we make use of the term micro level in the paper which refers to data management issues and macro level denoting guideline, policy, and legal governance concerns.

**Fig 1:**
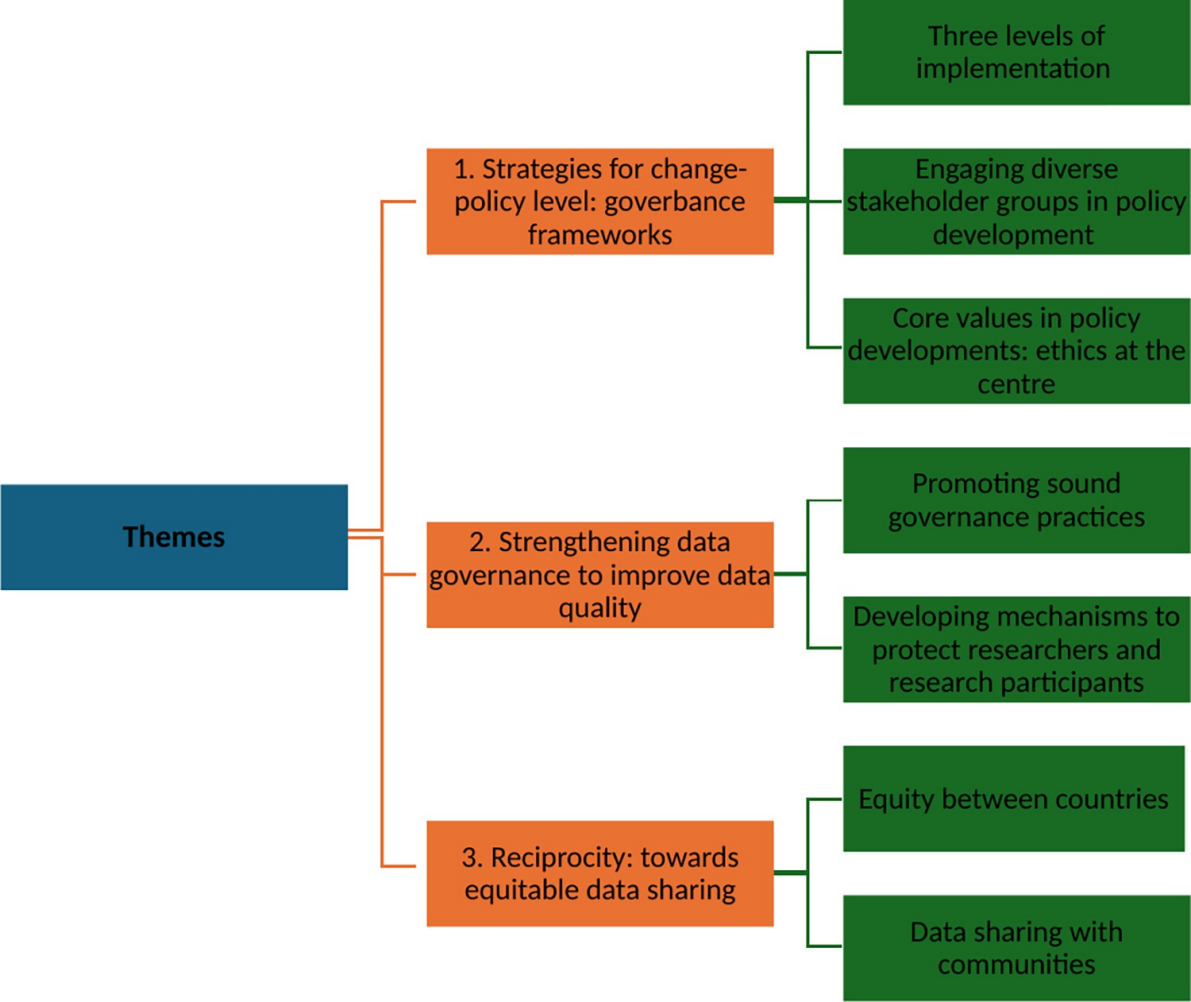
Visual representation of the three themes and subthemes.

The first theme focused on opportunities for change at a policy level specifically regarding the development of a guideline or framework to promote data sharing. Three sub-themes emanated from this theme, namely, three levels of policy implementation, engaging diverse stakeholder groups in policy development and core values in policy development: ethics at the centre. The second theme is referred to as strengthening data governance to improve data quality and includes two subthemes, promoting sound governance practices and developing mechanisms to protect researchers and research participants. Reciprocity towards equitable data sharing is the last theme which comprises two subthemes, equity between countries and data sharing with communities (Fig 1).

### Theme 1: Strategies for change at a policy level: governance frameworks

#### 1a). Three levels of policy implementation (institutional, international, national)

Lack of regulatory frameworks was recognized as a major barrier to data sharing for health researchers from SSA working in data intensive research [23]. Respondents thus articulated a need for a comprehensive guideline or framework to encourage data sharing amongst researchers in the SSA region. However, there was much discussion regarding the level of implementation this guideline should prioritize. In the extract below respondent 13 expressed that data sharing should be supervised and regulated by officials based at institutions, national departments, and international bodies.

“*… I think it is very important to all stakeholders including policymakers… we need such guidance at international level, at the national level, at the institutional level*…” (IDI 13).

Many respondents believed national laws should be prioritized due to the legally binding nature of a national law over a guideline proposed at an institutional level, with international influence.

*“International guidelines are good but what takes precedence is the national guideline because it is binding and in line with local available laws and regulations”* (IDI 9).

*“…I don’t think an institution can put in place a law or legislation—it will not be legally binding …when you look at the National Health Research System*, *one of the components are laws and legislation on managing data*. *So*, *for me countries should take that responsibility*” (IDI 5).

Naturally, this should be the government’s responsibility as this would fall under their mandate, as revealed by the excerpt below:

*“I would say it will have to be at a national level, international …could be adapted to our context so that we come up with something at national level and at institutional level… I think government should lead”* (IDI 12).

Respondents assigned explicit value to institutional guidelines especially the memorandum of understanding (MOU) in the context of collaborative research. However, respondent five below suggested that institutional and international guidelines should be developed in parallel with national guidelines and should be interpreted as an extension of national guidelines. This will significantly reduce uncertainties for researchers working within a multi-national research project.

*“… that research institutions are allowed in the spirit of partnership agreements …if it’s a multi country study to create a window for institutions to pursue by …. MOUs or agreements with other institutions, research institutions and share data within agreed principles. Each institution has that on the MOU—they can report to the National Councils of Science and Technology or National Health research organizations”* (IDI 5).

Policymakers should be cognizant of the significance of context when developing a framework for data sharing. Respondents two and nine below expressed similar views where researchers should be aware of existing laws and regulations of countries with whom they are sharing data.

*“…make sure that we look at the context of sharing. I mean the issue of consent cannot be overruled…in Ghana there are different rules operating here…the Data Protection Act. It might be different from Nigeria or South Africa or India …we cannot have generalized guidelines. Yes, we have to contextualize it so that in the end it will fit within the broader interest of what actually is happening within the specific country*” (IDI 2).

*“Whatever happens*, *the national guideline becomes the reference on what it is*. *For example*, *if I developed a guideline for Africa*, *do you think I will consider the unique aspects of South Africa*, *Zimbabwe*, *Uganda*, *Sudan*, *Algeria*, *Egypt*, *and so it’s very difficult*. *So*, *you make it generic*, *but then the country develops something more specific for its local setting”* (ID I9).

In addition, it is essential that policymakers ensure that when adapting a specific international guideline for use in a national context that the adaptation process is sound and considers all the critical contextual information of that specific country.

***“****For SSA…we don’t know which regulations apply across the borders. If I want to transmit data from Mauritius to, let’s say, South Africa. I don’t know whether there are the guidelines in South Africa and those that we have in [my country] now might be a generic guideline, but then it has to be adapted by country (*IDI 7).

Lastly all guidelines, should strive to promote the progression of researchers and society in general on the continent as stated below by respondent IDI 15.

***“****It must work for all people, in Africa, because this is the home base for where we are conducting the research. So, our rules of engagement must first and foremost work here …for the advancement of people on the African continent”* (IDI 15).

#### 1b) Engaging diverse stakeholder groups in policy development

*“You should have a diverse group of people on that committee”* (IDI 3).

This subtheme identified stakeholder groups that are essential in contributing to the development of a guideline promoting data sharing in SSA. Most respondents stated that a diverse group of stakeholders ought to form a committee where their input and specific expertise would be indispensable to assist policymakers with critically needed contextual information for a comprehensive and inclusive governmental data sharing guideline or data transfer agreement (DTA).

These various stakeholders include researchers, academic institutions, industries, lawyers, Information Technology (IT) specialists, ethicists, communities, research participants, international organisations, government departments, non-governmental organisations (NGOs), research ethics committees (RECs) and funders.

*“Multiple stakeholders—industries, government agencies, academia, research, institutions are involved. Of course, the communities, international key organizations and non-governmental organizations. I think at a policy level, our government-to-government collaborations for example, maybe under the African Union or at least at a level where countries sign up to some code of conduct”* (IDI 14).

***“****…The legal people should also be part of it…because at the end*, *if it becomes a policy*, *it has some sort of legal backing”* (IDI 2).

***“****And you should have an ethicist on that committee*… *you can ensure that the decisions being made by the committee are as ethical as they can be”* (IDI 3).

***“****But also*, *I will involve the Ethics Committee or Institutional Review Board”* (IDI 6).

*“Funders… in my knowledge*, *it is important to …involve them in this guideline but conflict of interest*?*”* (IDI 6).

*“People with computing background*, *because they’re the ones who are going to create the platform for sharing data”* (IDI 7).

*“And people from the community*, *especially those gate keepers and I mean*, *community engagement is the other issue that should be included in the guideline*. *CAB members*, *community advisory boards we have that*, *in our institute where we recruit representatives from the community who would advise us in research… not only in research but throughout the process”* (IDI 8).

#### 1c). Core values in policy development: ethics at the centre

Respondents consistently identified ethical principles and adherence to certain values within a research context as factors that required consideration when developing a guideline for data sharing. More specifically, values such as fairness, honesty, transparency, and integrity were recognized by researchers to be especially important to strengthen perceived notions of the societal benefits of research that reduce anxieties and fears regarding data sharing.

***“****Honesty is important. People should clearly indicate how they obtained the data”* (IDI 11).

***“****Openness and data integrity and integrity of the researchers and transparency”* (IDI 7).

*“I think transparency is also important for data sharing*. *If you make your data available to a wider audience … it increases trust in the people that we generate data from*. *The community where we generate our data*, *they are increasingly demanding transparency”* (IDI 14).

It was apparent from respondents’ accounts that acting in an ethical manner was accepted as upholding the core principles of research ethics, such as non-maleficence, beneficence and justice by respecting research participants and their rights to be well informed, providing consent, confidentiality, and anonymity. Placing ethics at the centre of the framework creates an enabling environment for research participants and researchers alike.

*“…You should prioritize non-maleficence. You should prioritize justice… and above all, most papers prioritize the overall good to society, but that’s without compromising any of the aforementioned. So, I think in those guidelines… we should look at providing an enabling environment for researchers to share data. Providing an enabling environment for benefits”* (IDI 10).

*“I’ll just speak of the principles of research ethics*, *where at the end of the day*, *the respect for persons will need to be integral to those values*. *I’ve already talked about beneficence*, *you know*, *the issues of nonmaleficence*, *justice*, *it’s important…the data sharing issues*, *we cannot really underscore the issues of confidentiality and data protection because data needs to be shared but again*, *researchers also need to think about the time invested”* (IDI 12).

***“****… at the top should be the protection of research participants…valid informed consent and protecting their confidentiality and*, *their privacy*: *the most risk for patients*. *These are critical issues that should be at the top of every guideline when it comes to health research”* (IDI 8).

### Theme 2: Strengthening data governance to improve data quality

It is imperative to explore issues at the data level and provide solutions to mitigate data-related challenges that researchers encounter. These challenges stem from fears around data protection, storage, data ownership, data validation, amongst others [23]. Data sharing will be seen as a viable option only when researchers or data users are encouraged to practice good data governance. Two sub-themes emanated from the second theme: 2a) promoting sound data governance practices and 2b) developing mechanisms ensuring protection for researchers and research participants.

#### 2a). Promoting sound data governance practices

Respondents underscored the need for researchers to practice good data governance which ensures that data is generated, organized, annotated, cleaned and corroborated in the correct manner. Data management is part of the process of data governance and raises awareness on issues involving data storage, data sharing, data monitoring and data regulations.

***“****Of course, you want high quality data. So, I’m making sure that the data is standardized, is cleaned, is validated and also make sure that there are good attributions of the data, making sure that the citations and acknowledgements are [done] properly, so that when it comes to benefit sharing, we know who was involved in generating what data”* (IDI 14).

*“So*, *by the time data needs to be shared*, *they would not be required to invest a lot of time to preparing the data for sharing because there is already a practice of proper data management”* (IDI 10).

***“****How long should it be retained*? *Who is responsible for archiving it*? *Issues around compliance with industry regulations and any other regulations that related to the data that is being generated… And I think some kind of monitoring of those data could also be important so that it’s not just garbage in and garbage out…trying to see how the data is maximally used increases in value while sharing it in an ethical manner”* (IDI 14).

*“So*, *the data sharing that’s a final component*, *but let’s talk data management… there’s lots of benefit to the data generator because that requires that you capture all the metadata required so*, *metadata management*. *Uh*, *getting to think about the storage*, *which means as you apply for grants*, *you can be able to also apply for grants to support data sharing*, *data management in itself*, *right*? *So that’s another benefit to them*. *The fact that you have your data well organized*, *then it means it will increase your productivity as a researcher”* (IDI 2).

***“****That should include ethical data sharing*, *consideration for benefit sharing*, *consideration for protection of human subjects and social harm*, *individual harm prevention*, *storage and storage standards and also maybe provide data repository requirements*, *including possibly having a national data repository… also include data security and privacy*. *Make sure we obtain informed consent*, *issues around what should be provided*, *intellectual property rights*. *Making sure that they understand the issues around IP registration for any innovation that is shared”* (IDI 14).

Good data governance ensures that data quality will increase data value. This in turn stimulates researcher productivity and growth.

A focus on adequate data management and promoting sound data governance practices will significantly contribute to researchers’ willingness to share data.

*“There is also a section on data sharing and data sharing agreements, whereby both the local institution and the collaborator, and another country should have equal rights to access that data to use it and to benefit from it”* (IDI 9).

*“OK and if we are to contribute to the guidelines…*. *For me*, *the very first thing to do is we need data protection acts to come into play because we don’t have that*. *Once we have the Act*, *maybe we are safe in terms of protecting our data…before this guideline can be developed*, *I want to see the data protection acts* (IDI 11).

Respondents nine and 11 further asserted that to practice good data governance, researchers need to be guided by regulations in the form of DTA or data protection acts. Moreover, respondent three mentioned that it would be ideal to have an oversight body or committee to monitor these acts independently, such as the REC.

*“…preferably the Ethics Committees or it should be coordinated by independent bodies”* (IDI 3).

#### 2b). Developing mechanisms to protect researchers and research participants

Respondents in the study spoke about their own vulnerability as confirmed in the companion paper [23]. Fears about scooping, publication pressure, and inadequate reward systems were deemed by researchers to be core factors for their reluctance to participate in data sharing [23]. To remedy the situation and provide researchers with some assurance, respondents suggested that mechanisms should be developed to protect them and their research participants.

Firstly, it is important to frame data sharing in a way that enhances the benefits to make it more appealing to researchers.

***“****I was quite involved in the drafting of our policy within the organization and there the critical component is getting to talk to the scientists and look at it from their perspective to ensure there is a very clear pathway towards data sharing. Framing it in such a way that there is very clear benefit to them”* (IDI 10).

Secondly, researchers need to continuously feel in control of their data. This assurance can be facilitated by having researchers as part of the data management process.

*“…mechanisms that would allow fair data sharing and one of the key aspects is if someone is going to access and use that data, they would probably get permission from the investigator who conducted the research before they can use it just like we all do for secondary data analysis, we always get back to the investigator and then ask for their approval before that”* (IDI 9).

Thirdly, researchers ought to be provided with sufficient time to adequately prepare high quality manuscripts for publication–prior to data sharing. This can be achieved by placing certain types of data under embargo.

*“… researchers need to be protected …they need time to really reap the fruit of working on that study and they must publish… the data protection issues are very much important. Let other people access their data once the key investigators think they’ve answered the key questions and let people acknowledge this data”* (IDI 12).

*“We needed those mechanisms in place and then of course we could have probably incentives for this research as well as collect the data to do the work first to utilize their data in publishing and maybe informing policy and probably developing products*, *before they share it*, *… maybe two years*, *maybe three years and after three years then the data can go to the open data source so that other researchers can benefit from it”* (IDI 9).

Lastly, researchers, research participants and data collectors should be adequately rewarded for their time and effort.

*“And they should definitely acknowledge the patients and the people who have actually collected the data and all that’s very important”* (ID I 7).

Constructive steps to obviate researcher and participants’ apprehensions will lead to solutions that ultimately increase data sharing where all parties feel as if they are benefiting from research findings and outputs.

*“…. So, I understand that science is a process, and everybody fits into the bigger scheme of things…there should be a bit more respect for the beginning of the pipeline and all the processes, particularly those that are involved with engaging directly with a human subject at the onset of a study. Because I think all of us place a lot more emphasis on, well, what can you do with the data, you know, what can you get out of it but there’s not much appreciation and recognition for the processes at large*” (IDI 15).

### Theme 3: Reciprocity: towards equitable data sharing

The last theme that was identified focused largely on an approach that may be instrumental in fostering data sharing amongst researchers in SSA and consists of two subthemes, equity between countries and data sharing with communities. The concepts of benefit sharing, recognition, reward, and capacity building are central to establishing an equitable data sharing approach.

#### 3a) Equity between countries

Respondent 14 below spoke about the importance of examining existing guidelines such as the Nagoya protocol to guide researchers to develop mechanisms for benefit sharing in their respective countries. This can be crucial in formulating researcher standards needed to ensure equitable data sharing.

*“… something like the Nagoya protocol, I would say that people who use data must be cognizant of and acknowledge in concrete ways, the people that have generated the data. I think we need more interventions like the Nagoya Protocol that tries to make concessions for benefit sharing. I think we need to have standards around benefit sharing so that every researcher, similarly to how you do your good clinical practice standards certification. I think we need standards around data sharing, and everyone adheres to good data sharing practices, as the basic minimum for all researchers”* (IDI 14).

To advance equitable data sharing respondent 15 articulated that actively striving towards reducing inequities that researchers and research participants face in the global South needs prioritization. The first step toward equitable data sharing for all in SSA will be achieved by recognizing the value of not only the data but all people involved in the research process—thus emphasising research integrity, capacitating researchers, highlighting benefits derived from research and by communicating with research communities.

*“When we have published information, we’ve also tried to make it more user friendly for people in the community, particularly the San Council, to have access to that and in one instance, when we had a science paper, we actually went back into the Kalahari on the occasion of Heritage Day and shared the excitement of the publication. Uh, another instance where we got an award for the publication that was monetary, I gave half to the San Council and the other half to the PhD student who did the work”* (IDI 15).

#### 3b) Data sharing with communities

It is imperative that benefit sharing, especially in the context of genomic data, requires strategic planning and negotiations with all the stakeholders involved in a fair and ethical way to facilitate the goal of equitable data sharing on the continent. This was reflected in the extract below.

“… *there should be mechanisms for benefit sharing right from the community where the research is done. They should need to be able to share from… how the derivatives are devolved will also talk about consent from the individuals who provided the data, we need to adequately obtain consent and tell them that their data is going to be shared, particularly genomic data. You know, data which may be identified and linked to community”* (IDI 9).

Furthermore, respondent nine expressed that in the context of benefit sharing, all parties involved should be consulted. It is vital that communities should not be overlooked in this process as this will hinder any chances of equitable data sharing.

“*Benefit sharing, it’s very, very important, to the community, to our local research settings, and then the international community, because research is done in a collaborative manner, all those different stakeholders need to benefit. We need to adequately share that”* (IDI 9).

Questions that need to be addressed at the outset when conducting research in communities within the context of benefit sharing, ought to explicitly specify the role of and benefit to each member. Respondent 15 below spoke about the importance of ethics in community engagement and how it is crucial for the researcher to define boundaries and stand firm in their beliefs to protect research communities to ensure that they ultimately benefit from the data that they have provided.

*“What is the investment from each partner and it has to be of mutual benefit. Very often when you’re working on the African continent, you know everybody wants you because they want access to your samples or your data that you’re generating because of the populations and the richness of diversity of genomes on the continent…I value my ethics and the respect of the communities who have given me the privilege to work among them. So, you get different people even among the research community and basically you as an individual need to define the parameters of your operation. And where I was concerned, it was very high and, and that is why I can still keep my head up high that I would never succumb to pressure. I worked with what was available and I made sure that from the very beginning I set the rules of engagement with would be collaborators or research partners*” (IDI 15).

## Discussion

The parent research project sought to determine the perspectives of health researchers in SSA in terms of data sharing management and governance to contribute to the development of data sharing guidelines in SSA. The quantitative survey obtained a broad overview of researchers’ perspectives from SSA countries. Part of the qualitative study focussed on challenges faced by SSA health researchers from 16 different SSA countries working with data intensive research [23]. This paper has deliberated upon respondents’ comments regarding opportunities to share African research data within regional settings and beyond.

Across the different themes and subthemes, participants were mainly in agreement. More specifically in respect of the first theme, “strategies for change at a policy level: governance framework”, participants agreed that there needs to be a comprehensive framework to encourage data sharing. However, more discussion and variability occurred regarding the level of implementation that this guideline should prioritise. Congruence in terms of responses were observed for subthemes 1b) engaging diverse stakeholder groups in policy development” and 1c) core values in policy development: ethics at the centre. For themes 2 and 3, participants were largely in agreement.

The challenges expressed and the strategies proposed to address them mirror the duality embodied in the concept of data governance as “the exercise of authority and control over the management of data” [33]. The “exercise of authority” at a macro level is embedded in guidelines, laws and policies to govern data at multiple levels in its lifecycle [33]. At the same time, data governance at a micro level has the potential to enhance data quality by improving resources for data management and by reducing data related expense and risk [33]. Abrahams et al (2019) describe six data decision domains that are imperative to consider when focusing on data governance: data quality, data security, data architecture, data lifecycle, metadata and data storage/infrastructure [33]. Progress has been made to strengthen data management and training on the continent [34]. More specifically, the Committee on Data international science council (CODATA, 2024, 1) strives to “promote global collaboration to improve the availability and usability of data for all areas of research” [35]. A strategic priority of CODATA focuses on data skills and building capacity for open science. Hence, CODATA has organised many workshops intended for researchers to develop and strengthen their data skills needed to ensure proper data management practices [35].

During data collection for this study, it was revealed that researchers from SSA are just as willing and determined to share data via regulated platforms. Their desire to attain high research quality as well as global recognition has instilled in them the importance of organizational structures that control data sharing without stipulating that researchers relinquish control of their own data [13]. At the core of improving data quality lie significant financial and human resources to manage data at a micro level. Such resources are often lacking in SSA. This is a pre-requisite for data sharing [36,37]. There are current initiatives geared towards data science in Africa for researchers to assist with data management such as the Data Science for health discovery and innovation in Africa (DS-I Africa) [38]. DS-I Africa is an initiative that strives to support the development of the required expertise among African scientists and to initiate and foster networks of African investigators [38]. The core vision of the initiative “is to create and support a robust pan continental network of data scientists and technologies that will be equipped to apply advanced data science skills to transform health” (DSI-Africa, 2022) [38].

Another future programme with a recent funding call is the data governance for Africa Initiative by Wehubit [39]. More specifically, this initiative “supports projects that use digital technologies in exploring new ways to solve development challenges with human centric economies in Africa working on regulation, cross-data border flows and green digital infrastructure” (Vota, 2023,1) [39].

SSA researchers in this study were emphatic that *stringent repository management* [15] was fundamental to their participation in collaborative health research where any form of data sharing occurred. Respondents conceded that responsible research data sharing can produce fundamental health benefits not only for researchers but also for primary respondents or participants, as well as the relevant institutions and sometimes their countries [4]. They valued data sharing opportunities for the myriad potential digital health benefits while they also called attention to their concerns in terms of management and governance when African researchers share data.

**Fig 2:**
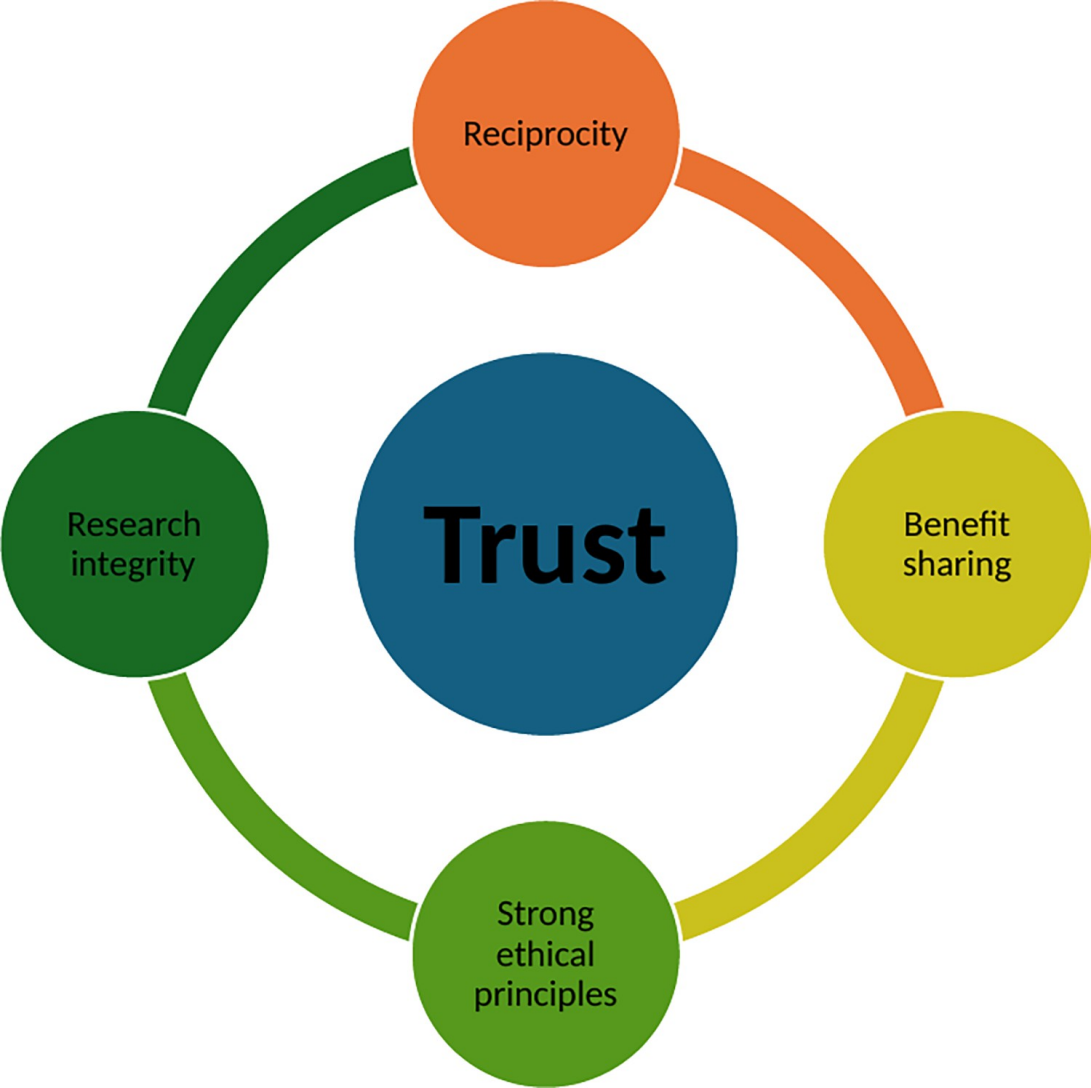
Visual representation of Trust and elements that are needed for equitable data sharing.

It was established that respondents were positive about data sharing opportunities if there was access to rigid, meaningful guidelines, completed and signed agreements as well as MOUs specific to that study. Although they emphasized various stipulations including the significance of data protection legislation and DTAs to secure their data or their interests in the data sharing process, the general opinion was that research data sharing is progressive and allowed various stakeholders recognition only if there were honest, trustworthy sharing processes [40,41]. While laws and DTAs are necessary requirements for good governance, they may not be sufficient to promote open data sharing. Research integrity and trust are therefore central to data sharing practices (Fig 2).

It is imperative to ensure that trust is built with research participants, researchers, and research communities. The concept of trust and its importance within research, clinical settings and diverse fields has been widely reported [42–44]. Researchers in South Africa have also turned their attention to trust and data sharing within the context of biobanking [45–47]. Literature from the field of biobanking has illustrated that when developing and strengthening trust with diverse stakeholders, it is critical to adhere to strong ethical practices [45,47,48]. Researchers in SSA in this study explicitly spoke about the importance of ethics as a contributing factor to fair data sharing practices. Hence, it is important to look at ethical decision-making frameworks that are consistent with philosophical thinking in SSA [49]. Sambala et al. (2019) explored the merits of looking at public health research and decision- making based on the philosophy of Ubuntu. “Through its emphasis on humanity, compassion, and social responsibility, Ubuntu (“I am because we are”) has the potential to facilitate solutions to and avert conflicts between individual rights and public health” [49]. By shifting the focus from traditional western notions of moral reasoning and decision-making to a more African approach allows for a deeper understanding of practices and needs of research participants, their communities and researchers in the SSA region [49–51] have illustrated the utility of framing genomics research within the moral theory of Ubuntu and using it as a foundation to address inequity and advance ethical principles such as justice, fairness, solidarity, open sharing, and mutual trust.

Benefit sharing helps to build trust. The respondents interviewed stressed that privacy was just as important to the process of sharing as was making data available to all. They stated that effective decision making during the data sharing process could lead to benefits for all stakeholders either financially or professionally [1]. Benefit sharing has been recognised [2,52] to be an important mechanism to guarantee a balanced and fair distribution of research derived outputs. While there are recognised guidelines such as the Council for International Organisations of Medical Sciences (CIOMS) [53] and the Nagoya Protocol on Access and Benefit Sharing [54], the detrimental impact of the vast inequities between countries from the Global North and South and particularly the lack of bargaining power of LMIC stakeholders is often neglected [10,55]. Haelewaters et al., 2021 discuss ten core rules to tackle power imbalances faced by Global South researchers. This includes establishing equal partnerships and mutually beneficial collaborations, actively initiating Global South collaborations, focusing on establishing synergistic collaborations, complying with local written and unwritten rules, acknowledging and embracing differences in working culture, inculcating equitable and collaborative research practices early on, use of local infrastructure, integrating a capacity building element, establishing ethical and fair practices regarding publications and authorship and ensuring research is made accessible through local dissemination [10].

An example of a successful case of benefit sharing in South Africa is the rooibos benefit sharing agreement between the South African San Council and the South African Department of Environmental affairs (DEA) [56]. This was a groundbreaking agreement where an indigenous group such as the San were able to take control of their traditional knowledge and derive monetary benefits with possible future requirement of non-monetary benefit sharing as well [56]. Core principles such as respect, honesty and care were underlined during the agreement process [56]. This is in line with the communitarian philosophy that Ubuntu embraces [49].

Similarly, Bedeker et al., (2022) have proposed a framework that focuses on advocating for a more ethical approach to benefit sharing for researchers and stakeholders from LMICs and under resourced areas. The framework is essentially a practical tool to aid users to recognize opportunities for benefit sharing within research programmes and is based on the socioecological model. The framework is divided into two dimensions, the goal of the first dimension is to identify stakeholders, dimension two shifts focus to defining how benefit sharing can be implemented [52].

Macpherson (2019) argues that in LMIC’s there is a need to strengthen capacity building, and this should be viewed as an extension of benefit sharing. In addition, the role of context and power imbalances has been recognised as playing a crucial role in attempts to level the playing field for researchers from LMICS [55]. Respondents in the study highlighted that within the policy development sphere, paying attention to context is critical in developing a more ethical approach to data sharing. Moreover, Macpherson (2019) has argued that identifying and addressing disparities between key stakeholders such as the community members, research participants, researchers, the public and institutions is vital in encouraging trusting research relationships [55]. Funders, such as the National Institutes of Health (NIH) in the United States, which strongly support data management, sharing and governance in Africa (Data Science for Health Discovery and Innovation in Africa), play a crucial role in this regard [38]. However, the limitations of the funding landscape should also be considered. Resnik (2018) has argued that while funders have an obligation to distribute health resources based on expectations related to contribution to social value and the public good, the commitments of different funders may differ based on diverse philosophical assumptions or politics [57].

A study that assessed research data management (RDM) capabilities at a university in Ghana revealed that RDM is still lagging yet there is enormous potential for growth [58]. Similarly, findings from a study conducted in SA with researchers from 26 public universities revealed low levels of data literacy (DL) training [59]. Moyo and Bangani (2023) recommended that librarians and research support personnel should play a crucial role in developing training needs and strengthening DL amongst researchers [59].

Open, transparent researchers as well as ethical data sharing processes and guidelines will inevitably lead to empowering research collaborations. At the same time researchers on either side of the data sharing process are obliged to maintain confidentiality for the duration of the research and during sharing. In conjunction with confidentiality, primary respondent identities and personal characteristics are to remain anonymous to protect them [1–3]. Researchers, especially within the health sector, are encouraged to engage in data sharing. Onus is upon the primary researcher to obtain informed consent to participate in the study and allow for recording and sharing of data. Studies have demonstrated that transformation in healthcare data capturing systems and rapid AI development have resulted in the demand for more proficient data sharing systems in high income countries (HICs) [60–64]. Shah et al. (2022) applied a cross-sectional survey within four countries and advocated for data sharing to be aligned from the initial point of the face-to-face interview with the patient [63]. However, they emphasize the importance of maintaining equilibrium between privacy factors with the ultimate healthcare system and data sharing benefits. In their study, Li et al. (2022) conducted a systematic review of Electronic Health Records in HICs. They concluded that there was a higher prospect of meticulously captured clinical data evident from these studies where successful systems applied data quality algorithms to avert errors. Hence, such high quality of data completeness and planning generated by HICs increased efficiency of automatic data capturing and sharing; for example, the use of e-fax data [60]. However, such highly sophisticated systems ensuring de-identification, appear to be the prerogative of HICs. Health information technological advances and AI demand that health data be captured more efficiently electronically within secure network systems [61]. With the privilege of affordable fully digitized systems, HICs have the advantage over LICs and MICs. Expedient integration of data incentivizes researchers and promotes fair, de-identified data exchange within the health systems and data sharing among researchers. Improved Healthcare Systems, properly regulated data capturing and sharing frameworks and data collection policy modifications may be promoted via leading-edge enlightened political and professional contributions creating proficient collaborative partnerships between healthcare and society [61,62,64]. Seastedt, et al. (2022) also verified that with AI and advanced ‘global medical knowledge systems’ came greater interest in data sharing because of improved governance and with it a decrease in re-identification [62].

Ultimately, research integrity lies at the heart of building trust in research collaborations. Bak et al., (2023) explored the concept of governance of health data in research and has advocated for an approach that prioritizes trust and mutual cooperation between researchers and research participants [48]. In their paper, the authors argue that while data protection legislation holds obvious benefits, researchers can feel restricted by the laws imposed and can view DTA’s as stifling the scientific process [48]. To counteract the possibility of harmful consequences of data protection policies, Bak et al., (2023) have proposed practical solutions where trust is the cornerstone of this framework [48]. More specifically, their conceptual agenda explores three main ideas of trust, namely, trust as a multi-agent belief, encompassing the values of rationality and democracy and viewing trust as a means for priority setting [48]. This is consistent with findings of our study where researchers have recommended that while it is important to draft laws and regulations that promote data sharing and establishes data security what is equally important is instilling core ethical values as well as developing trust between researchers, research participants and communities to truly harness the benefits of data sharing for all parties involved.

### Strengths and Limitations

Although this study yielded rich data on strategies to promote data sharing in SSA, many Francophone and Lusophone countries are not represented. This is due to the research being conducted by an English-speaking team, the lack of French and Portuguese speaking researchers in SA with expertise in data-sharing and the high costs of translation of transcripts from other languages to English. Obtaining 16 IDIs took nine months of concerted effort with challenges related to internet access, lack of responses to email invites and connectivity issues. Thus, aiming for a bigger sample size would require several more months. The aim was not to reach saturation per country but to obtain an overall picture of mitigating challenges experienced by health researchers from different SSA countries. We have completed an in-depth study in one country—South Africa—with a manuscript to be submitted for review shortly. So, data saturation was reached from a continental perspective in that respondents from different countries expressed similar views by interview 16 but country saturation in the wider SSA region was not reached. This is an area for further research in different countries. We have expanded the qualitative enquiry in SA in this manner and the same could be done for other countries. Despite these limitations, the data obtained is valuable and data saturation was reached after sixteen interviews.

## Conclusion

Efficient management structures and policies, based on robust legislation, will encourage data sharing in SSA. However, trust in research collaborations and a culture of strong research integrity supersedes the security provided by laws and guidelines. Data sharing practices will be strengthened if ethics is acknowledged as a key contributing factor and by utilizing approaches such as Ubuntu to address instances of inequity or exploitation that plagued researchers in the past and hindered data sharing. Likewise, improving data management at a micro level to enhance data quality is non-negotiable. However, it is important to be cognizant of the role that funders and research support personnel play in strengthening data management. An inclusive approach involving multiple diverse stakeholders is critical in the development of guidelines and tools for data sharing. Benefit sharing was also identified as a key concept in facilitating data sharing. Successful initiatives such as the rooibos benefit sharing agreement provides evidence that reciprocity amongst stakeholders is core to building trust and that an equitable approach to data sharing holds immense utility and benefits for all involved.

## Supporting information

S1 File Interview Guide. (DOCX)
