# Supplementary material for: Trust as moral currency: Perspectives of health researchers in sub-Saharan Africa on strategies to promote equitable data sharing
Source: PLOS Digit Health. 2024 Sep 27;3(9):e0000551. doi: 10.1371/journal.pdig.0000551 (PMC11432837; doi:10.1371/journal.pdig.0000551)
Supplement: S1 File — (DOCX) [file pdig.0000551.s001.docx]

**S1 File**

**Interview Guide for Data Scientists**

Prior to the interview, a study investigator will provide an overview of the research purpose and will remind the participant of the confidentiality and anonymity measures adopted in the research. The study investigator will obtain permission to record the interview. Participants are free to decide whether their camera remains on or is switched off during the recording of the interview on the following virtual platforms, namely Zoom, Microsoft Teams, or Skype.

**Interview Themes**

1. Introduction focusing on participant’s position/function in their institution.

- What is your professional/scientific background?
- What is the significance of the type of data you use or generate?

1. Participant’s understanding of data sharing and previous experience thereof.

- What are your thoughts on sharing your data on the various available platforms?
- What are your thoughts on sharing others’ data?
- What would encourage you to make your data available (if not already available)?

1. Participant’s opinion concerning the promises and challenges of data sharing.

- What hurdles do you face in collecting your research data?
- What do you think about the benefits of data sharing?
  - Are the benefits sufficient and/or worthwhile?
  - Do all parties involved benefit?

1. Existing guidelines and criteria adopted to handle data sharing-related issues

- Do you follow any specific guidelines to assess data sharing?
  - If yes, which ones?
- Are you aware of guidelines from national or international organisations?
- Do you have the sole responsibility of approving access to your data?
- If no, who does?

1. Participant’s suggestions to develop an inclusive data sharing guideline policy.

- If you could contribute to the drafting of new guidelines, what would you suggest?
- Which values would be most important?
- Who do you think should develop such guidelines and at which level (international vs. national)?
